# The acquisition and measurement of surface waves of high-speed liquid jets

**DOI:** 10.1007/s12650-015-0307-9

**Published:** 2015-08-14

**Authors:** Chen Gong, Minguan Yang, Can Kang, Yuli Wang

**Affiliations:** School of Energy and Power Engineering, Jiangsu University, Zhenjiang, 212013 China; Department of Mechanics, KTH-Royal Institute of Technology, 10044 Stockholm, Sweden

**Keywords:** Surface wave, High-speed microscopic photography, Image processing, Wavelength measurement, Breakup regime

## Abstract

**Abstract:**

The instability analysis of the liquid jet issuing into ambient air was conducted with an emphasis placed upon the evolution of surface waves of the jet. An experiment was designed to visualize the microscopic morphology on the surface of a liquid jet. A spectral method was proposed to measure wavelength from the obtained jet images. We also discuss key setup parameters that significantly affect the resolution of desired jet features and the accuracy of the spectral measurement. The results show that the liquid jet near the nozzle exit can be divided into a laminar section, a transition section, an instability section, and a turbulence section. Surface wave scales range from 0.06 to 0.11 times of the nozzle diameter with the atomization breakup regime. For the atomization breakup regime, the growth ratio of the surface waves of the instability section is 0.06 which is 1.5 times the value of the second wind-introduced breakup regime and 3 times the value of the first wind-introduced breakup regime.

**Graphical abstract:**

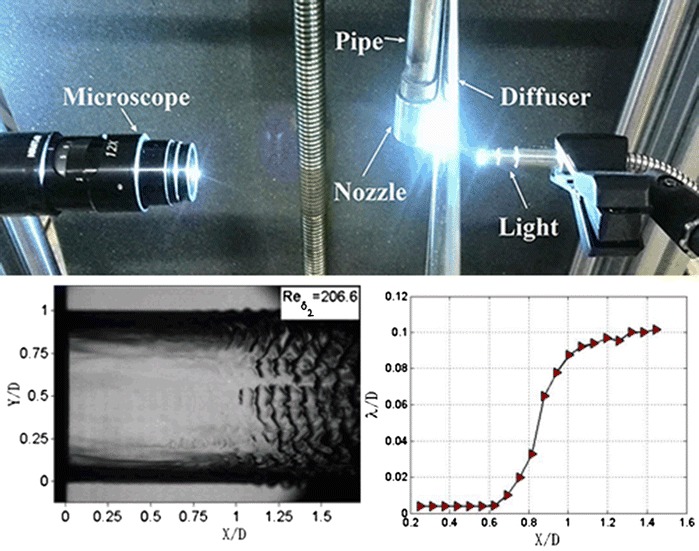

## Introduction

Break up of liquid jet is a common phenomenon and it has been widely used in many fields. For example, the atomization process plays a critical role in combustion systems since most power generation processes necessitate liquid fuel. The purpose of injector design is to produce droplets of given size, distribution, and velocity under certain environment. The combustion efficiency and pollutant emission depend greatly on spray characteristics. However, our understanding on jet breakup process is still not adequate.

After a liquid jet discharges from a nozzle, surface waves are anticipated on the jet surface due to the boundary condition as the jet surface shifts from a non-slip boundary condition to a free surface boundary condition. These surface waves will be strengthened by the interaction between the jet and the ambient fluid. The development of these surface waves causes liquid jet breakup and droplets are finally produced (Lefebvre 1989). Based on Ohnesorge number and Reynolds number, the breakup of the liquid jet can be divided into four different regimes: Rayleigh breakup, first wind-induced breakup, second wind-induced breakup and atomization (Chigier and Reitz 1996). Lin and Reitz (1998) have proposed the expressions of the four regimes based on the Weber number. Atomization regime occurs at Weber numbers larger than 40.3. The jets with this regime break up near the nozzle exit and the diameters of the droplets which pinch off from the jets’ surface are much smaller than the diameters of the jets. Some research suggested that the formation of the droplets was the result of the development of the jet surface waves.

To explore the physical essence of jet breakup, the development of the surface waves should be granted a priority. Some researchers studied surface waves via numerical simulation. Representatively, Shinjo and Umemura (2010, 2011), Ménard et al. (2007), and Fuster et al. (2009) had depicted the development of surface waves that lead to atomization and captured the ligaments and droplets from the disturbed liquid core surface. Numerical simulation requires initial and boundary conditions which are pre-determined by either stability analysis of different degrees of complexity or experimental measurements. The existing stability analysis cannot explain all the physical mechanisms of the high-speed liquid jet.

Many researchers endeavored to study the development of the surface waves using experimental methods. Some of them are dedicated to small jets of diameters less than 1 mm. Yon et al. (2003) and Li and Collicott (2006) performed visualizations of the liquid jets using coupling shadowgraph and laser sheet techniques. Paciaroni et al. (2006) developed the ballistic imaging technique which makes use of the ultra-short laser pulse to image the near-field liquid core. The coupling of ballistic imaging and X-ray absorption imaging was used by Briggs et al. (2006) to investigate high-speed jet primary breakup. The high rate imaging technique and the very high spatial resolution imaging technique were developed by Delacourt et al. (2005) and Nakagawa et al. (2006), respectively. In Aliseda’s experiment (Aliseda et al. 2008), the camera was focused through a microlens on a small region located at the outlet of the liquid nozzle, thereby, the micro-structures of capillary jet could be obtained. Blaisot and Yon (2005) used image-based techniques to describe the shape of liquid particles that are not fully atomized or relaxed.

The others consider the behavior of large jets with diameters exceeding 1 mm. Osta et al. (2012) investigated the surface topography of liquid jets using X-ray diagnostics. With the help of Shadowgraph, Mayer (1994) and Mayer and Branam (2004) captured the surface waves and measured the wavelengths using traditional approaches. The pulsed photograph and holograph were used by Faeth et al. (1995), Wu and Faeth (1993, 1995), and Wu et al. (1992, 1995) to observe breakup characteristics at jet surface, Sallam et al. (2002) completed the works of Wu and Faeth, and examined the breakup of the entire liquid column for turbulent liquid flows. In Hoyt and Taylor’s experiments (Hoyt and Taylor 1977a, b), a relatively large nozzle was used and the backlight was employed. Jets with bright surface waves were produced. Moreover, surface wavelength measurement based on nine still images of their jet was provided and various stability analyses (Yoon and Heister 2003; Park 2005) validated by this wavelength. The aforementioned works provided several useful methods for further research works.

In this paper, a visualization experimental method is designed to capture the surface waves of high-speed liquid jet. The important parameters of the visualization experimental method are discussed. The velocities of the liquid jet range from 12 to 38 m/s. Spectral methods were used to process the captured jet images. Two commonly used spectral methods and their key parameters were discussed. Furthermore, the relationship between jet surface waves and Reynolds numbers is investigated based on quantitative information extracted from jet images. The vigorousness of the new method devised here to capture small-scale morphological information is highlighted and such a method is expected to be extended to other applications.

## Experimental approach

### Setup

**Fig. 1:**
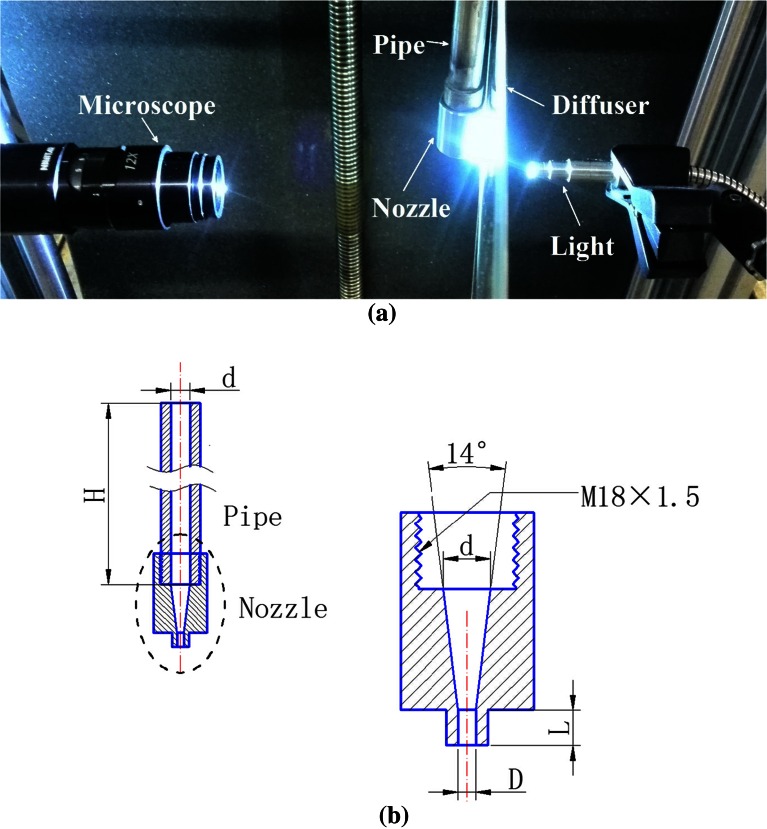
Components of the experimental system: **a** on-site image; **b** schematics of the nozzle and the pipe

The experimental setup is presented in Fig. 1. Water is pumped from a container, the flow rate of the pump is controlled by gearing a variable-frequency drive, and the jet velocity is changed accordingly. The flow condition is produced with a straight stainless steel pipe of length *H* = 0.5 m and inner diameter *d* = 8 mm. The length-to-diameter ratio of the straight stainless steel pipe exceeds 40, ensuring fully developed inner turbulent flex (Wu et al. 1995). The pump and the straight stainless steel pipe are connected with a rubber hose. The nozzle is made of copper and consists of two parts, contraction section and straight section. The inlet diameter of the contraction section match the upstream pipe’s inner diameter and the angle of the contraction section is 14°. The contraction section is followed by a straight section with diameter *D* = 3.0 mm and stretches to a length of *L* = 6.0 mm.

Jet images were captured with an OLYMPUS I-SPEED 3 camera and a microscope. The exposure time and the frame rate of the camera were set as 1 μs and 100 fps, respectively. For each experimental condition, 100 images were captured to constitute a complete sampling group. The image size of the camera is 1280 × 1024 pixels. The amplification of the microscope is set as 2.8 which provides typical resolution around 6 μm/pixel. The light is produced by OLYMPUS ILP-2 and transmitted through an optical fiber with a diameter of 6 mm. The light beam passes through a 5-mm-thick acrylic diffuser plate before touching the jet surface. The acrylic diffuser plate is used to produce a uniform light distribution. The light, liquid jet, and microscope are collinear.

The jet velocity near the exit of nozzle is measured by particle image velocimetry (PIV). The PIV system consists mainly of a YAG200-NWL pulsed laser, a POWER-VIEW 4MP CCD camera, and a macro lens. Water temperature is measured using a WS-T11PRO digital thermometer; density and kinematic viscosity are determined accordingly.

The flow in the straight section of the nozzle is treated as a flat plate flow (Hoyt and Taylor 1977a). The momentum thickness *δ*_2_ at the exit of the nozzle can be calculated by the laminar Blasius solution for a flat plate:

1 $$\delta_{2} = 0.664\sqrt {\frac{\nu L}{U}},$$

where *ν* is the kinematic viscosity, and *U* is the jet velocity.

The Reynolds number based on the momentum thickness can be calculated by

2 $$Re_{{\delta_{2} }} = \frac{{U\delta_{2} }}{\nu }.$$

The Weber number based on air density can be calculated by

3 $$We_{g} = \frac{{\rho_{g} U^{2} D}}{\sigma },$$

where *ρ*_g_ is air density, *σ* denotes surface tension.

The flow parameters and physical properties in our experiment are presented in Table 1.

**Table 1:** Flow conditions

Parameter	Value
Nozzle diameter (*D*)	3.0 mm
Liquid temperature (*T*)	293 K
Kinematic viscosity (*ν*)	1.007 m^2^/s
Liquid density (*ρ*_L_)	998 kg/m^3^
Ambient pressure (*P*)	0.1 MPa
Air density (*ρ*_g_)	1.21 kg/m^3^
Jet velocity (*U*)	12.0–38.2 m/s
Momentum thickness (*δ*_2_)	8.35–14.9 μm
*Re*_*δ*2_	177.7–316.7
Surface tension (*σ*)	0.07275 N/m
Weber number based on air density (*We*_g_)	7.21–72.77

The accuracy of the digital thermometer (WS-T11PRO) used in our research is ±0.5 °C. Therefore, the uncertainty of the measured liquid temperature is 2.5 %. The liquid density, kinematic viscosity, and surface tension are estimated based on the liquid temperature. Based upon literatures (Bian et al. 2010; Syuto et al. 2010; Hori and Sakakibara 2004), the uncertainty of velocity measurement is less than 3 % with a confidence level of 95 %. The quantities of Reynolds number *Re*_*δ*2_ and Weber number *We*_g_ can be calculated from their relationship with the above quantities. Taking account of error propagation, the accuracy of the *Re*_*δ*2_ and *We*_g_ is less than 4.5 and 6 %, respectively.

**Fig. 2:**
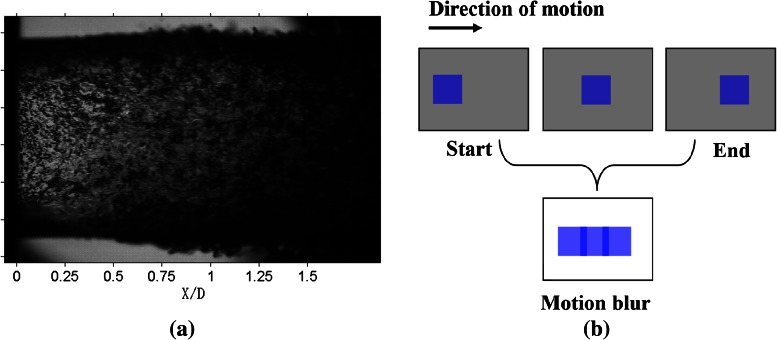
Motion blur. **a** A blurred jet image. **b** The schematic of motion blur

To capture the micro-structures of the high-speed liquid jet, experiment must provide sufficient temporal resolution. The exposure time of the camera must be short enough to capture the instantaneous jet profile, otherwise motion blur will take place, as shown in Fig. 2a. Motion blur can be briefly explained by Fig. 2b; the jet is denoted by the square inside the rectangular windows. And the exposure time (Δ*t*) is equally divided into three smaller moments (1/3 Δ*t*). If the jet moves in an appreciable distance during the exposure time, namely, the position of the square varies within exposure time, which can result in motion blur. As can be seen in Fig. 2b, the three squares will not overlap in the last image, and that is the so-called motion blur. In this case, the exposure time should be reduced to 1/3 Δ*t* to avoid motion blur. In principle, for continuous motions, the exposure time should be infinitely small. However, the distance that the camera can discern is limited. Therefore, if the distance covered by the jet within the exposure period is smaller than the distance that the camera can distinguish, the motion can be ignored and motion blur can be avoided accordingly. The minimum distance that can be distinguished by the camera used is 46 μm. Based on the maximum jet velocities exhibited in Table 1, the exposure time should be less than 1.1 μs. In this experiment, the exposure time is set to 1 μs.

Also there should be sufficient spatial resolution to resolve the surface waves. Based on the theory of Portillo, surface wave size scales with the momentum thickness (Portillo 2008). The momentum thicknesses vary from 8.1 to 14.9 μm; therefore, the spatial resolution of the jet images should be no less than 8.1 μm/pixel.

**Fig. 3:**
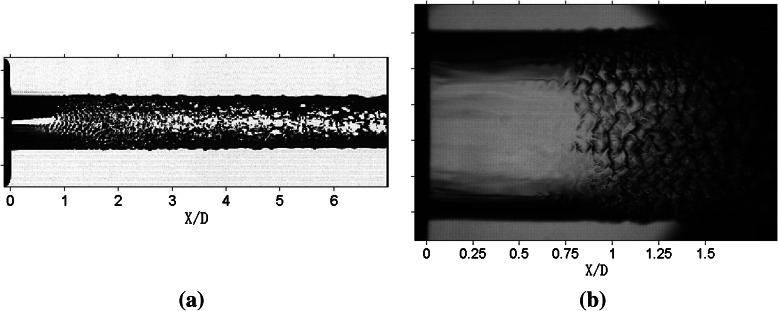
Jet images of different spatial resolutions. The spatial resolutions of **a** and **b** are 50 and 6 μm/pixel, respectively

Jet images with different spatial resolutions are presented in Fig. 3. The two jets are produced under the same experimental condition. However, compared with Fig. 3a, the image in Fig. 3b is focused through a microscope on a smaller rectangular region (about 6 mm × 7 mm). In Fig. 3b, distinct structures, such as “fish skin”-like structures can be observed. Moreover, it can be seen that the surface waves are distributed along the streamwise periodically. But in the left picture, the surface waves appear to be blurry and the degree of order is rather low.

### Image processing

**Fig. 4:**
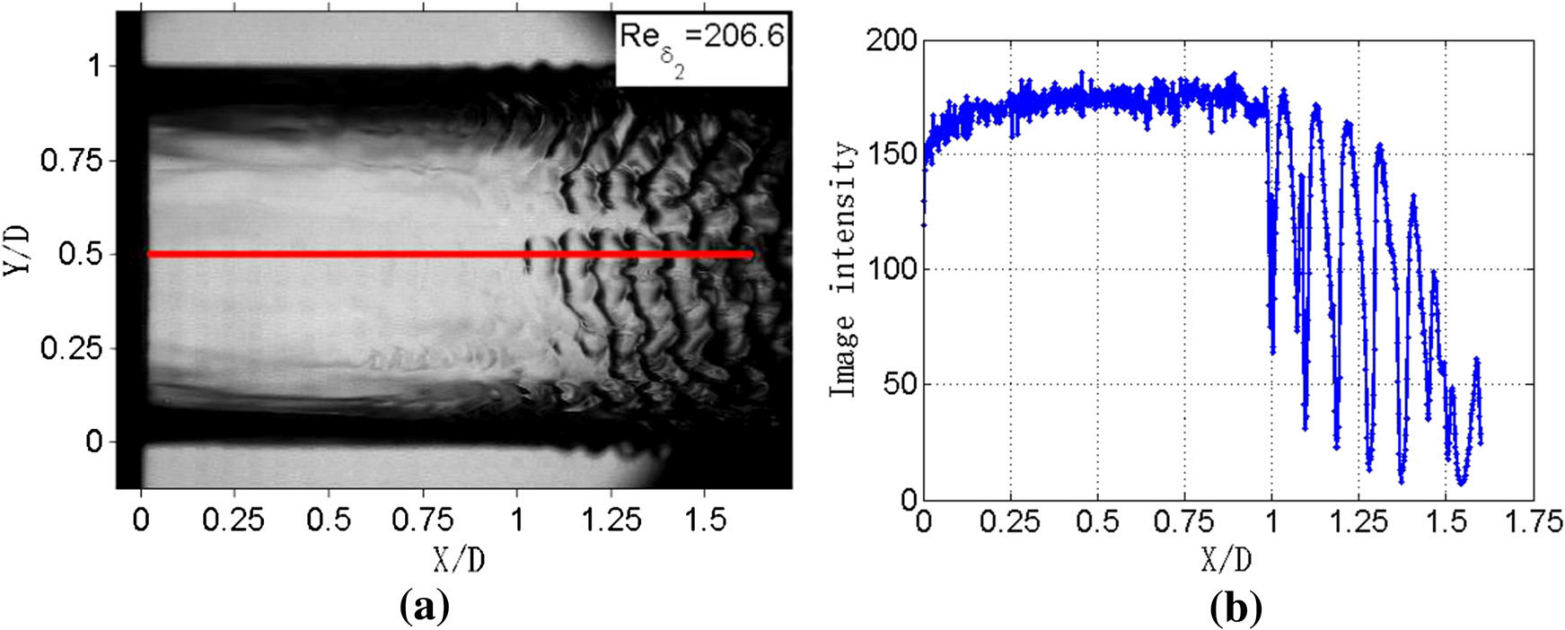
Jet image with *Re*_*δ*2_ of 206.6. *X* is the streamwise direction; *X* = 0 is the orifice of the nozzle exit. *Y* is the spanwise direction, *Y* = 0 and *Y* = 1 are the upper periphery and lower periphery, respectively

Detailed information of the jet surface waves can be measured by processing the image intensities of the pixels of the jet images. As shown in Fig. 4a, a red line extends from the nozzle in the streamwise direction. The corresponding image intensity distribution along this line is presented in Fig. 4b. Firstly, in the *X*/*D* range of 0–1.0, the curve of the image intensities fluctuates violently and it has small amplitude. Normally, such small difference of image intensities can hardly be perceived with naked eyes. Compared with Fig. 4a, it is easy to find that this part is corresponding to the smooth part of the jet. Then the curve has significant change near *X*/*D* = 1.0. The curve of this part has obvious periodicity. It has significantly bigger amplitude and lower frequency compared to the former part. The streamwise lengths between adjacent wave crests are about 0.09 *D* which is equal to the distance of adjacent surface waves in Fig. 4a. The development of the surface waves along the streamwise direction can be well represented by the image intensities. That is to say, the jet surface waves can be well measured by processing the digital signals extracted from the jet images.

Jet images are constructed based upon digital information representing image intensities. The digital information essentially reflects morphological characteristics of the free surface. Two spots on the surface that have similar morphological structures will have similar values of image intensities. Therefore, we can use power spectral density (PSD) estimation to analyze the periodicity of image intensities variation and then calculate the wavelength.

Welch method is one of the commonly used digital signal processing methods (Mulgrew et al. 2002). The computational formula of the Welch method is expressed as follows:

4 $$\bar{P}\left( w \right) = \frac{1}{MUL}\sum\limits_{i = 1}^{L} {\left| {\sum\limits_{n = 0}^{M - 1} {x_{N}^{i} \left( n \right)d\left( n \right){\text{e}}^{ - jwn} } } \right|}^{2},$$

where *N* is the length of the sample, *L* is the number of the segments, and *M* is the length of each segment. $x_{N}^{i} \left( n \right)$ is the individual dataset. $d\left( n \right)$ is the window function. $U = \frac{1}{M}\sum\nolimits_{n = 0}^{M - 1} {d^{2} } \left( n \right)$ is a normalizing factor used to achieve an asymptotic and unbiased estimation. ${\text{e}}^{jwn} = \cos \left( {wn} \right) + j\sin \left( {wn} \right)$ is the complex sinusoidal; *w* is the circular frequency. $\sum\nolimits_{n = 0}^{M - 1} {x_{N}^{i} } \left( n \right)d\left( n \right){\text{e}}^{ - jwn}$ is the Fourier transform of random sequence of segment *i*, 1 ≤ *i* ≤ *L*.

According to the principle of the Welch method, the result of the Welch method depends on the selection of window function. The measurement of jet surface waves is conducted to obtain the main wavelength. So the main lobe signal should be highlighted and the side lobe signal should be suppressed. The Blackmann window is one of the trigonometric function windows. It has relatively high side lobe attenuation rate boosting the concentration of spectrum energy on the main lobe. Therefore, the Blackmann window is selected.

Along the streamwise direction, a series of measurements are performed. The first sample is taken from the exit of the nozzle and the length of the sample is set equal to the window length. The measured result is set as the wavelength of the middle point of this sample. Then the second sample was taken from the position of 0.06 *D*, approximately 33 pixels, downstream of the nozzle exit. The length of the second sample is equal to the first sample. The procedure was repeated until the end of the data set.

**Fig. 5:**
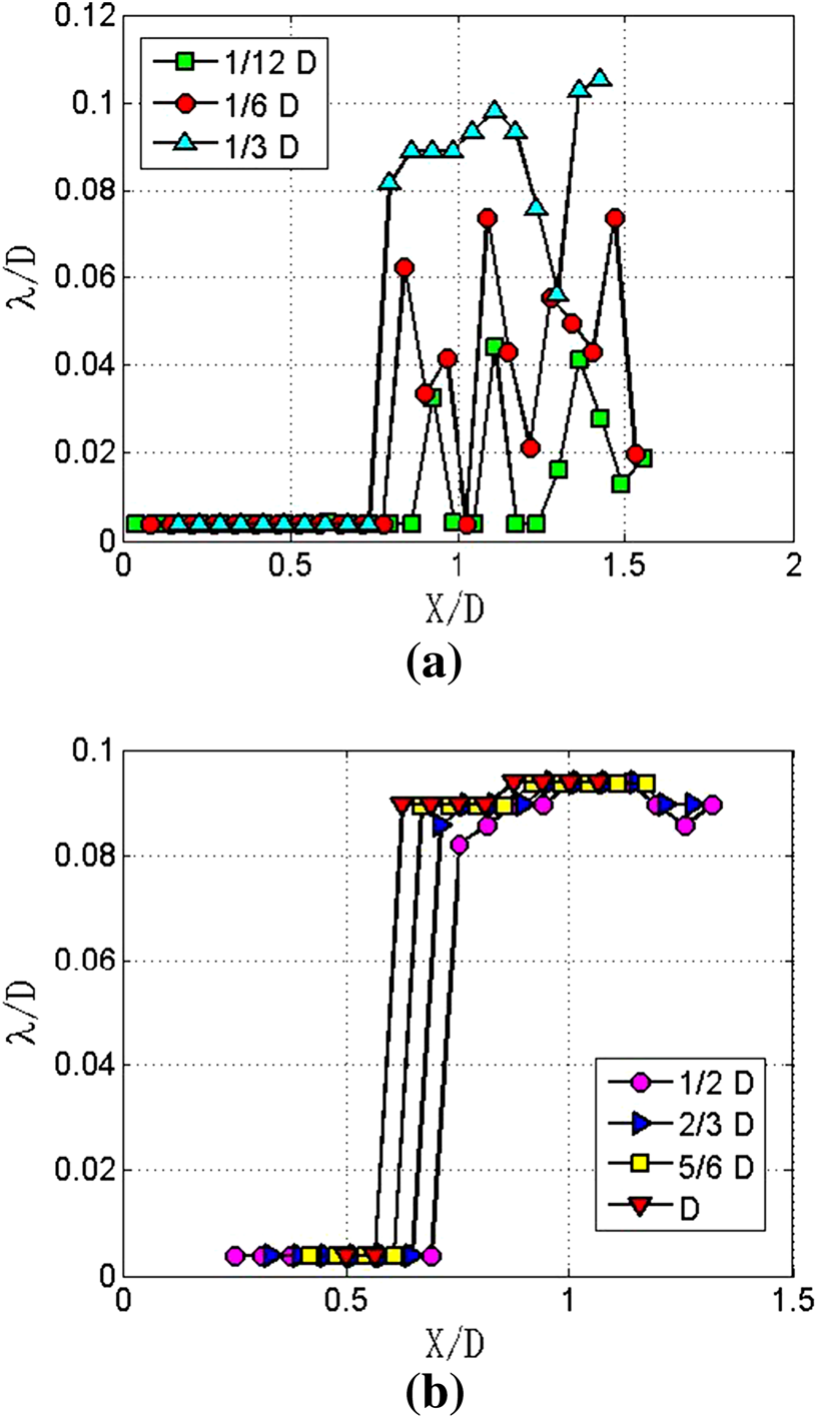
Wavelengths measurement with different lengths of window function. **a** Window lengths range from 1/12 D to 1/3 D. **b** Window lengths varies from 1/2 D to D

In order to get appropriate length of the window, jet images are processed with different lengths of windows. Take the image intensities of Fig. 4b for example. The results are presented in Fig. 5. These curves can be generally divided into two parts along the streamwise direction. In the forepart, the wavelengths are all the same and smaller than 0.01 *D*. In the back part, the wavelengths are considerably bigger than forepart. Particularly, in Fig. 5a where the window length is smaller than 1/3 *D*, the wavelengths fluctuate violently. However, in Fig. 5b where the window length is larger than 1/3D, the wavelengths are nearly coincided and are around 0.09 *D* which is equal to the value of the streamwise lengths between two adjacent wave crests in Fig. 4b. Clearly the window length should be larger than 1/3 *D*. However, as window length increases, the streamwise range of wavelength measurement decreases. As seen in Fig. 5b, for the window length of *D*, the streamwise range is only limited within *X*/*D* = 0.5–1.1. So the window length is set as 0.5 *D*.

Take a further look at Fig. 5b, when the length of window is equal to 1/2 *D*, the measured wavelength suddenly increases at the position near the *X*/*D* = 0.75. The corresponding sample was taken from *X*/*D* = 0.5–1. The measurement shows that the transition position from the smooth part to the part incorporating surface waves is located at about *X*/*D* = 1, as is in agreement with the results in Fig. 4.

Another typically digital signal processing method, Burg method, is also considered. This method does not require window function. The computational formula of the Burg method is

5 $$P_{x} \left( {{\text{e}}^{jw} } \right) = \frac{{\sigma^{2} }}{{\left| {1 + \mathop \sum \nolimits_{k - 1}^{P} a_{k} {\text{e}}^{ - jwk} } \right|^{2} }},$$

where *P* is the degree of model $k = 1,2, \ldots p$, the reference value of *P* is $N/3 < P < N/2$, *N* is the length of the sample. $\sigma^{2}$ is white noise.$a_{k}$ is a coefficient. ${\text{e}}^{ - jwk} = { \cos }\left( {wn} \right) - j\,{ \sin }\left( {wn} \right)$ is the complex sinusoidal, and *ω* is circular frequency.

**Fig. 6:**
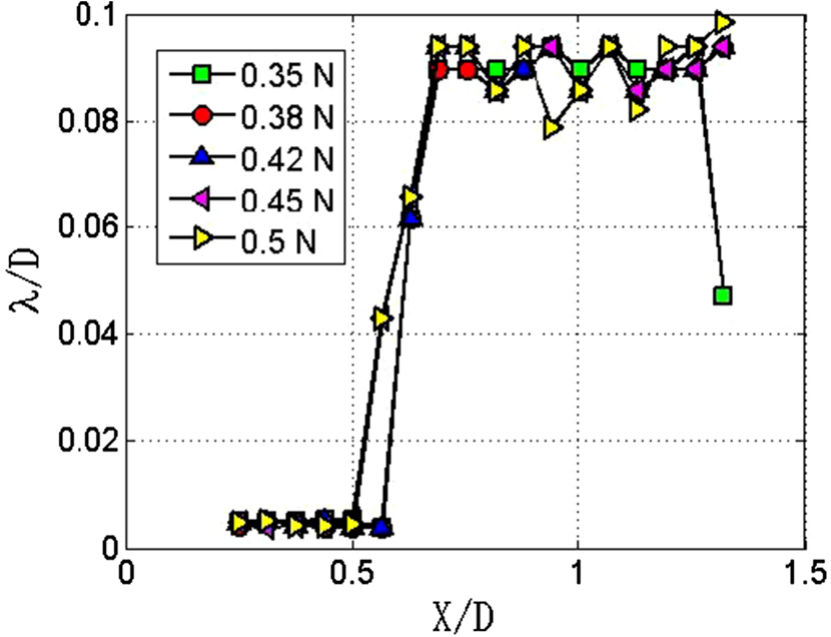
Wavelength measurement with Burg method of different *P*. The length of *N* is 1/2 *D*

The key parameter of Burg method is the degree of model *P* (Portillo 2008). Using Burg method with different *P* values to process the same image intensity data of Fig. 4a, the results are presented in Fig. 6. Within the *X*/*D* range of 0.2–0.6, the wavelengths are nearly the same and are less than 0.01 *D*. The wavelengths fluctuate around 0.09 *D* within the *X*/*D* range of 0.6–1.4. The position at which wavelength significantly increases is about *X*/*D* = 0.6, and the corresponding transition position is located at about *X*/*D* = 0.85. Clearly, the transition position measured by Welch method is more accurate, so the Welch method is selected for jet image processing in the presented study.

Based on the analysis above, the Welch method and Blackmann window were selected to process the jet images. The length of the window function was set as 0.5 *D* which corresponds to 260 pixels. To reduce the random error, spanwise positions between *Y* = 0.25 and *Y* = 0.75 were measured, and then, all the other 99 jet images which were captured under the same experimental condition were processed by the same method. Take the average value of these 100 images as the final wavelengths.

**Fig. 7:**
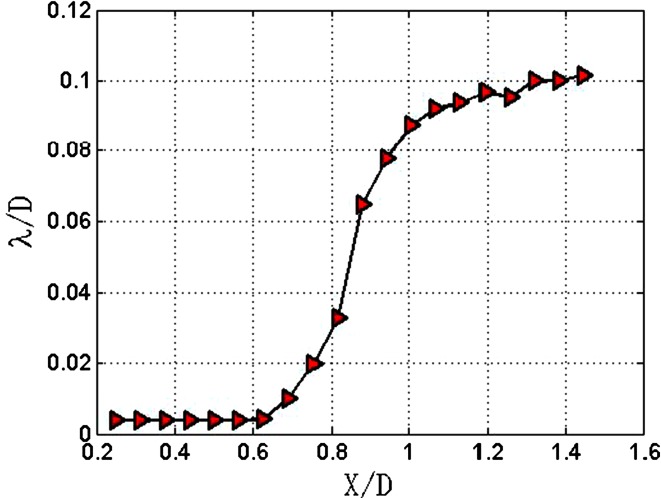
Average wavelengths of *Re*_*δ*2_ = 206.6

The average wavelengths of the jet with *Re*_*δ*2_ equal to 206.6 are presented in Fig. 7. The results can be divided into three parts. Firstly, within the range of *X*/*D* = 0.25–0.6, the wavelengths are all the same and equal to 0.004 *D*. Secondly, within the *X*/*D* range of 0.6–1, wavelength increases with a slope of 0.23. Thirdly, the slope of the curve drops to 0.04 in the *X*/*D* range of 1–1.45.

**Fig. 8:**
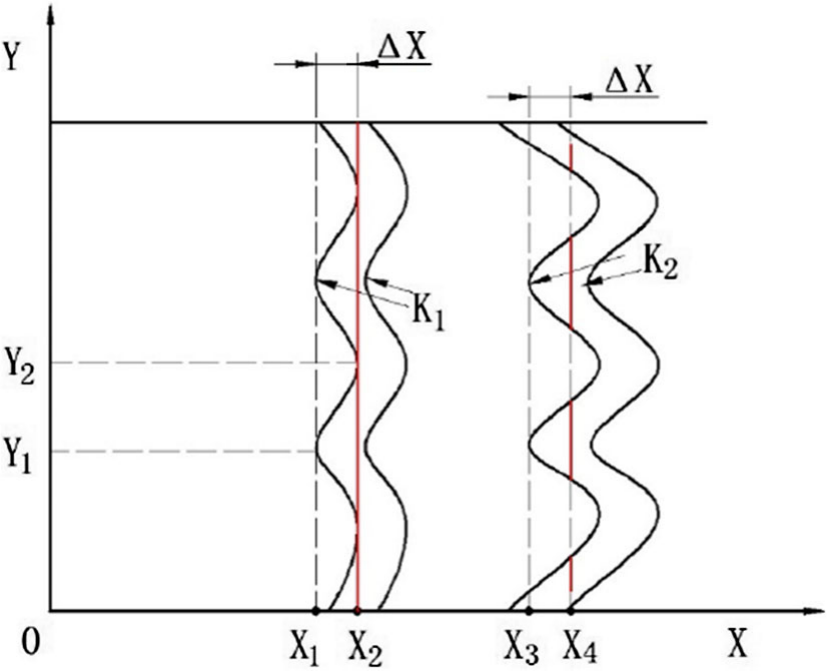
Analysis of difference between single measurement and multiple measurements. *X* represents the streamwise direction; *Y* represents spanwise direction

In Fig. 7, wavelength increases with a slope of 0.23 in the streamwise range of *X*/*D* = 0.6–1. However, wavelength goes up vertically at the streamwise position of *X*/*D* = 0.75, as shown in Fig. 5. The obvious disparity between the two measured results can be explained, as shown in Fig. 8.

As depicted in Fig. 4a, along the spanwise direction, the surface waves of the jet are not straight lines but a group of curves with various curvatures. In Fig. 8, the arc segments are used to represent surface waves. Clearly, surface waves are different at different spanwise positions. For example, the sample taken from the spanwise position of *Y*_1_ first reaches the surface waves at the streamwise position of *X*_1_. However, for the spanwise position of *Y*_2_, the sample first reaches the surface wave at the streamwise position of *X*_2_. From *X*_1_ to *X*_2_, the length through which sample can reach the surface waves is increased. That is to say, average wavelength is increased along the streamwise direction. This explains why there is a difference between the averaged result and the single result.

Take a further look at Fig. 8; it is apparent that surface wave curvature has a great influence on the growth ratio of wavelength. There are two groups of surface waves in Fig. 8 with equivalent wavelengths but different curvatures. The surface waves relatively close to the origin have smaller curvature of *K*_1_. The length between *X*_1_ and *X*_2_ is equal to Δ*X* which is the same as the horizontal distance between *X*_3_ and *X*_4_. The red line segments represent the increment of the spanwise range that sampling can reach the surface waves as the sample moves in streamwise direction with a distance of Δ*X*. Clearly, the surface wave with smaller curvature has an increased length. In other words, the surface wave with smaller curvature has a larger wavelength growth ratio.

### Validation

In this section, the accuracy of the Welch method in image processing was validated. The validation was carried out based upon the examination of two parameters: wavelength and transition position.

**Fig. 9:**
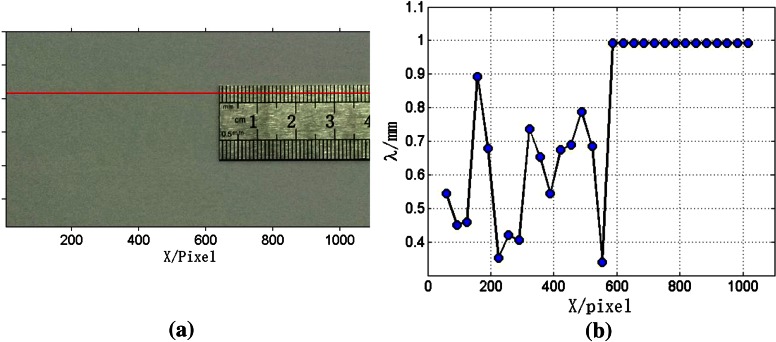
Validation with a ruler image. **a** Rule image. **b** Processing result

Figure 9a exhibits an image of a ruler, the image intensities of the pixels located on the red line in Fig. 9a were processed. These image intensities were divided into two parts. In the range of *X* = 0–640 pixels, the image intensities were extracted from the background which has no explicit order. The image intensities of *X* = 640–1090 pixels were based on the ruler image, and the distance between the two adjacent calibrations is equal to 1 mm which corresponds to 11.6 pixels. The length of the window was set as 118 pixels which was decided with the aforementioned method. The distance between every two neighboring samples was set as 33 pixels.

The processing result was presented in Fig. 9b. In the range of *X* = 0–590 pixels, the wavelengths have no order and fluctuate from 0.3 to 0.9 mm. However, in the range of *X* = 590–1090 pixels, the wavelengths are all the same and equal to 0.99 mm. The error of the wavelength measurement is 1 %. At the position of *X*= 590, wavelength significantly increases and corresponding samples were taken from *X* = 531–649 pixels. That is to say, the measured transition position is located at *X* = 649 pixels. The error of transition position measurement is about 1.4 %.

## Results and discussion

### Structures of liquid jets

**Fig. 10:**
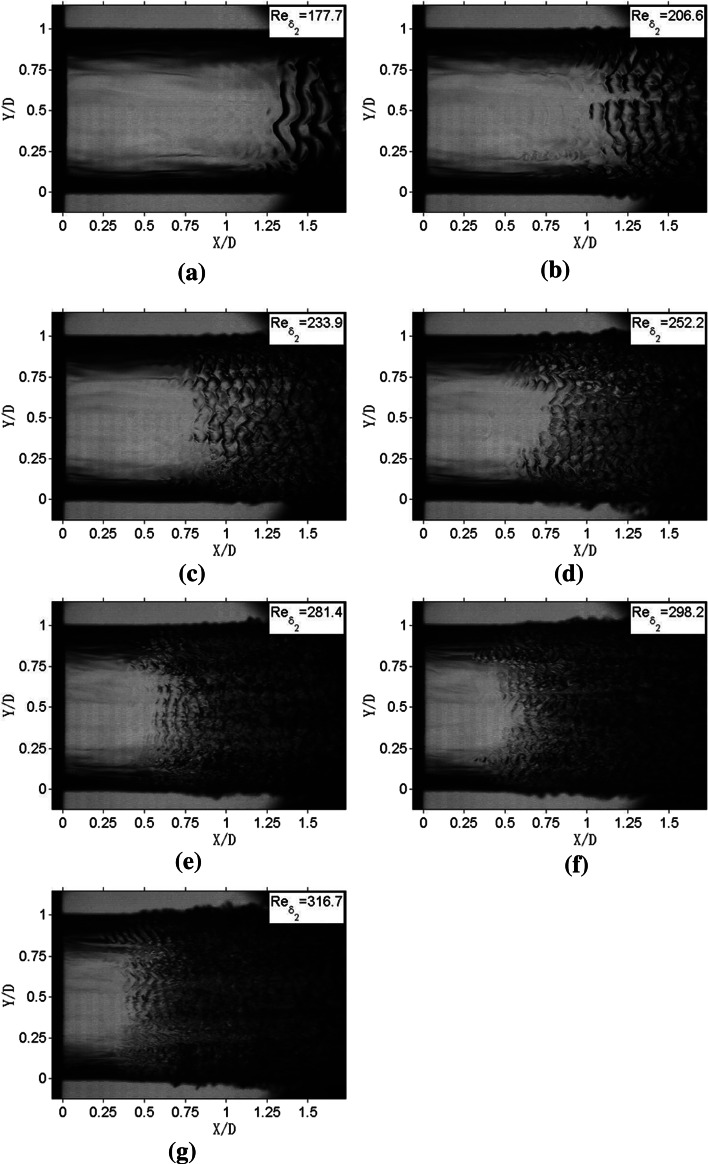
Surface waves of jets with different *Re*_*δ*2_

The surface waves of the liquid jets with different *Re*_*δ*2_ values are presented in Fig. 10. *Re*_*δ*2_ values in Fig. 10 vary from 177.7 to 316.7 and *We*_g_ changes from 7.21 to 72.77 accordingly. Based on Lin’s theory (Lin and Reitz 1998), the breakup regimes of these jets cover first wind-induced breakup regime, second wind-induced breakup regime, and atomization regime.

The jet in Fig. 10a falls into the first wind-induced breakup regime. The jet can be briefly divided into two parts. Firstly, within the streamwise range of *X*/*D* = 0–1.25, it has a nearly smooth surface. The upper periphery and lower periphery of the jet are straight lines. The diameter of the jet has no change in this part. Here the parts with smooth surface are defined as the laminar section. Secondly, at the streamwise position of *X*/*D* = 1.25, there is a group of structures featured by distinct waves. Along the spanwise direction, the configuration of these surface waves is apparently of sine-curve type, and the distance between crests and troughs is about a quarter of the diameter of the jet stream. Along the streamwise direction, these surface waves have remarkable periodicity: (1) the surface waves have the same configuration; (2) the distances between two adjacent surface waves are nearly all the same and equal to 0.12 *D*. The parts that have periodic surface waves are defined as the instability section.

The jets in Fig. 10b–d are compatible with the second wind-induced breakup regime. Similar to Fig. 10a, the jet of Fig. 10b can also be divided into laminar and instability sections. However, the instability section in Fig. 10b is different from that shown in Fig. 10a. Firstly, the surface waves in Fig. 10b arrive at the streamwise position of *X*/*D* = 1 approaching further to the nozzle exit. Secondly, the distance between adjacent surface waves is about 0.1 *D*, which is smaller than that shown in Fig. 10a. Thirdly, along the spanwise direction, surface waves in Fig. 10b are also in the shape of the sine curves, but the scales of these sine curves are smaller than its counterpart in Fig. 10a.

The surface waves in Fig. 10c are significantly different from the surface waves in Fig. 10a, b. On one hand, in the spanwise direction, the surface waves in Fig. 10c look like fish skin instead of sine curves. On the other hand, for different spanwise positions, the distances between two adjacent surface waves are different. For example, at the spanwise position of *Y* = 0.4, the distance is about 0.1 *D*, however, at the spanwise position of *Y*/*D* = 0.6, the distance is 0.07 *D*. In addition, only within the streamwise range of *X*/*D* = 0.75–1.25,the surface waves have periodicity. In the part downstream of *X*/*D* = 1.25, the interaction of the surface waves with different scales makes the structure of the surface waves more complex and unordered. The parts that only have unordered surface waves are defined as the turbulence section.

As for Fig. 10d, the streamwise positions at which surface waves appear are different at different spanwise positions. At the spanwise position of *Y*/*D* = 0.5, the surface waves arise at the streamwise position of *X*/*D* = 0.75. However, at the spanwise positions of *Y*/*D* = 0.25 and *Y*/*D* = 0.75, the surface waves come out at the streamwise position of *X*/*D* = 0.6.

The jets in Fig. 10e–g are in the atomization regime. For this regime, transition from laminar to turbulence is accelerated. First of all, the distance between adjacent surface waves in Fig. 10e is about 0.06 *D*, which is only the half of the corresponding value for the jet of the first wind-induced breakup regime. Secondly, the lengths of the laminar sections of the jets in Fig. 10e–g are less than 0.5 *D*, which are also smaller than those of the first wind-induced breakup regime. Moreover, for Fig. 10f–g, no instability sections are perceived and the jet surface transforms from the laminar section to turbulence section directly.

### Wavelengths

**Fig. 11:**
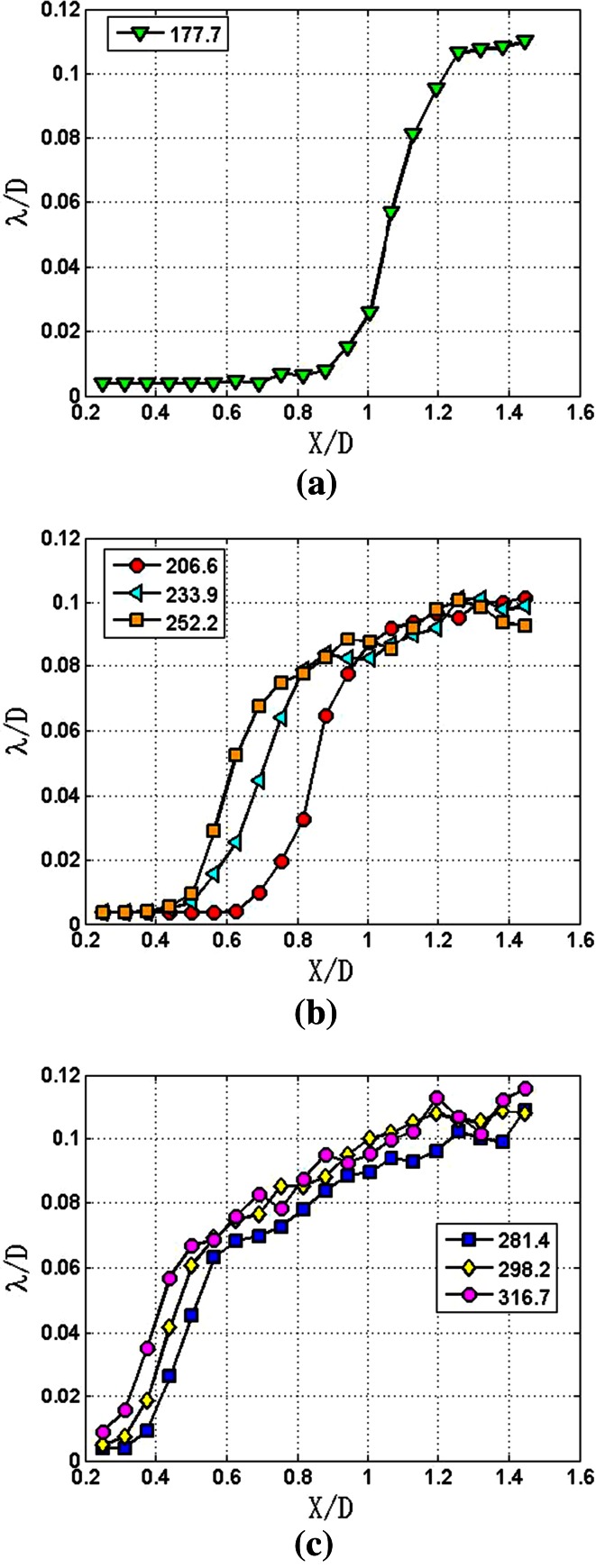
Wavelengths of different breakup regimes: **a** first wind-induced breakup regime, **b** second wind-induced breakup regime, **c** atomization regime

The measured wavelengths of different breakup regimes are presented in Fig. 11.

The laminar section of the jet under the first wind-introduced breakup regime has the longest length of 0.75 *D*. In the transition section, the wavelengths went up from 0.004 *D* to 0.106 *D* with a slope of 0.23. The length of the transition section is 0.5 *D*. The instability section starts at the streamwise position of *X*/*D* = 1.2. The wavelengths of this section have a slight increase from 0.106 *D* to 0.11 *D* with a relatively small slope of 0.02. The wavelengths of this breakup regime are comparable to the diameter of the nozzle.

In the second wind-induced breakup regime, the jets have short lengths of laminar section. With the increase of Reynolds number, the lengths of laminar sections decrease from 0.628 *D* to 0.376 *D*. The transition sections of these three jets have similar trends; the length and the slope of these transition sections are 0.4 *D* and 0.23, respectively. The instability sections of these jets are nearly overlapped, and the slope of these instability sections is 0.04 which is twice the value of the slope of first wind-introduced breakup regime. The wavelengths of the instability sections range from 0.08 *D* to 0.1 *D*. At the streamwise position of *X*/*D* = 1.3, the wavelengths begin to decline. According to this, it is concluded that the inception of turbulence sections occurs at this position.

Under the atomization regime, the lengths of the laminar sections are further reduced. Particularly, with *Re*_*δ*2_ of 316.7, the jet has no laminar section within the measurement range, which means the length of the laminar section is less than 0.5 *D*. The length of the transition section drops to 0.3 *D*, but the slope is still equal to 0.23. The instability sections range from *X*/*D* = 0.6–1.2, and the wavelengths of instability sections rise from 0.06 *D* to 0.08 *D* with a slope of 0.06. Clearly, the growth rate of wavelength of the instability section is the largest under the atomization regime.

## Conclusions

A visualization experimental method is designed to capture the surface waves of high-speed liquid jet. Two spectral methods were discussed and the Welch method was used to process the captured jet images. The main conclusions are as follows.

1. Based on surface morphology and the obtained wavelengths, the jet stream near nozzle exit is divided into four sections: laminar, transition, instability, and turbulence sections. The jet under the atomization breakup regime has the shortest lengths of laminar section and transition section of 0.4 *D* and 0.3 *D*, respectively, but the longest length of instability section of 0.6 *D*. All the transition section slopes of the three breakup regimes are 0.23.
2. In the first wind-introduced breakup regime, the lengths of surface waves of the jet are about 0.11 *D*. However, the wavelengths of the jet range from 0.08 *D* to 0.1 *D* under the second wind-introduced breakup regime. Moreover, the jet has a larger scale of wavelengths ranging from 0.06 *D* to 0.11 *D* under the atomization breakup regime.
3. The slope of instability section depends on breakup regime. Under the atomization breakup regime, the slope is 0.06 which is 1.5 times that of the second wind-introduced breakup regime and three times that of the first wind-introduced breakup regime.
